# In-Depth Characterization of Black Soldier Fly Larvae Reared on Phenolic-Rich Agro-Industrial Substrates

**DOI:** 10.3390/insects17030292

**Published:** 2026-03-06

**Authors:** Claudiu-Nicusor Ionica, Katalin Szabo, Bernadette-Emőke Teleky, Silvia-Amalia Nemeş, Rodica-Anita Varvara, Dan Cristian Vodnar, Călina Ciont, Alina Diana Haşaş, Mircea Coroian, Romelia Pop, Sorana Daina, Andrei-Radu Szakacs, Adrian Macri

**Affiliations:** 1 Department of Animal Nutrition, Faculty of Veterinary Medicine, University of Agricultural Sciences and Veterinary Medicine of Cluj-Napoca, Calea Manaștur, 400372 Cluj-Napoca, Romania; claudiu-nicusor.ionica@usamvcluj.ro (C.-N.I.); sorana.matei@usamvcluj.ro (S.D.); andrei.szakacs@usamvcluj.ro (A.-R.S.); adrian.macri@usamvcluj.ro (A.M.); 2 Institute of Life Sciences, University of Agricultural Sciences and Veterinary Medicine of Cluj-Napoca, CaleaMănăştur 3-5, 400372 Cluj-Napoca, Romania; bernadette.teleky@usamvcluj.ro (B.-E.T.); amalia.nemes@usamvcluj.ro (S.-A.N.); rodica-anita.varvara@student.usamvcluj.ro (R.-A.V.); dan.vodnar@usamvcluj.ro (D.C.V.);; 3 Department of Clinical and Paraclinical Sciences, University of Agricultural Sciences and Veterinary Medicine of Cluj-Napoca, 400372 Cluj-Napoca, Romania; alina.hasas@usamvcluj.ro; 4 Department of Parasitology and Parasitic Diseases, University of Agricultural Sciences and Veterinary Medicine of Cluj-Napoca, 400372 Cluj-Napoca, Romania; mircea.coroian@usamvcluj.ro; 5 Department of Pathology, University of Agricultural Sciences and Veterinary Medicine of Cluj-Napoca, 400372 Cluj-Napoca, Romania; romelia.pop@usamvcluj.ro

**Keywords:** alternative protein source, bioactive compounds, insect-based feed, nutritional and functional profile

## Abstract

Black soldier fly (*Hermetia illucens*) larvae (BSFL) represent a sustainable protein source for animal feed, efficiently converting organic waste into high-value biomass. This study aimed to valorize agricultural by-products (apple, potato, and red beetroot peels) as rearing substrates to obtain larvae enriched with bioactive phenolic compounds, while evaluating their nutritional, functional, and safety characteristics. Larvae were reared on diets with varying inclusion levels of each peel’s by-products. Proximate analysis showed that the substrate type and inclusion level significantly (*p* < 0.05) influenced larval composition, with consistently high protein and variable ash and fat contents. Colorimetric measurements indicated that phenolic-rich diets, particularly apple by-products, promoted cuticle darkening, reflecting the impact of dietary phenols on pigmentation. Functional properties were also modulated by the substrates: 2% potato peel yielded the highest phenolic content, while 20% apple peel produced the highest flavonoid concentration, both enhancing antioxidant capacity across CUPRAC-Cupric Ion Reducing Antioxidant Capacity, ABTS-2,2′-azino-bis 3-ethylbenzothiazoline-6-sulfonic acid, and DPPH-1,1-diphenyl-2-picrylhydrazyl assays. Rheological analysis confirmed desirable non-Newtonian, shear-thinning behavior, suggesting improved technological quality. Mycotoxin testing revealed low Aflatoxin B1 but variable Zearalenone levels, highlighting the influence of substrate composition on toxin metabolism. Overall, agricultural by-products can produce enriched BSFL with enhanced nutritional and antioxidant properties, as long as the substrate choice and inclusion levels are carefully optimized for safety.

## Simple Summary

Black soldier fly (*Hermetia illucens*) larvae (BSFL) are emerging as a sustainable and nutritious ingredient for animal feed and human food, offering a rich source of protein and fat. The larvae’s unique ability to thrive on diverse organic waste makes them key players in relation to the principles of the circular economy, transforming low-value agricultural by-products into valuable resources. This study investigated the nutritional and functional quality of BSFL using agricultural waste, specifically apple, potato, and red beetroot peels, as a special diet source. We found that the type and amount of organic waste used strongly affected the larvae’s composition in a non-linear way. Rearing the larvae on these by-products enriched their nutritional profile with phenolic compounds and flavonoids, which boosted their antioxidant capacity and further resulted in health-related benefits. Furthermore, the specialized diets altered the physical properties of the resulting biomass, improving its suitability for feed manufacturing. While this valorization strategy produces a superior, functional protein source, the study also identified a need for careful safety controls, as levels of certain mycotoxins varied significantly depending on the substrate. Overall, agricultural by-products are a promising way to obtain BSFL, offering a path to sustainable, high-quality animal feed and human food.

## 1. Introduction

Edible insects such as *Hermetia illucens*, known colloquially as the black soldier fly larvae (BSFL), belonging to the *Stratiomyidae* family, *Diptera* order, are increasingly recognized as sustainable ingredients for future food and feed systems [1]. The larvae have been established as an alternative to conventional livestock due to their high protein content (35–45% dry mass), lipid fractions (20–35% dry weight), and availability of essential amino acids, fatty acids, and minerals [2]. Additionally, their production requires fewer resources compared to traditional livestock, with up to 100 times less land and water use, resulting in a drastically reduced carbon footprint. BSFL are an increasingly popular ingredient in the animal feed industry due to their rich nutritional content and the ease of production. The use of BSFL-based ingredients is gaining popularity for a wide range of animal species like swine, laying hens and broilers, fish, dairy cows, pets, etc. [3,4]. It also constitutes an actual subject on novel food application, as dried, defatted BSFL powder is presently under review by the European Food Safety Authority (EFSA) for its use in human food products [3]. The larvae can be efficiently reared on organic waste streams, transforming these materials into high-value biomass, making them a cornerstone of sustainable protein production systems [4]. At the same time, it has been observed that the substrate used for rearing impacts the nutritional and functional profile of BSFL, influencing both the quality and the quantity of bioactive compounds [5].

Although most of the studies related to the use of substrates for improving growth performance of larvae focus on constituents such as proteins [6], there are also studies that highlight the use of certain ingredients in the rearing process to enrich the antioxidant capacity of larvae by stimulating the accumulation of phenolic compounds in the larval biomass [3]. Due to the larvae’s ability to develop on nearly any organic substrates, agricultural by-products are generally preferred as an economically and ecologically suitable choice. Additionally, utilizing agro-food by-products to obtain insect-based biomass supports the circular economy principles, particularly when by-products rich in bioactive compounds (e.g., phenols and carotenoids) enhance both sustainability and product value [3]. Among the vast array of agricultural by-products, potato peels (*Solanum tuberosum*), red apple peels (*Malus domestica*), and red beet peels (*Beta vulgaris*) stand out due to their remarkable nutritional profile containing dietary fibers, vitamins, minerals, as well as bioactive compounds [7,8,9].

According to FAO data, global potato production in 2023 was approximately 383 million tons, with China and India being the main producers, at 93.5 million and 60.1 million tons, respectively [10]. This volume of production is estimated to generate over 50 million tons of potato peel waste annually [11]. Similarly, the large-scale cultivation of beets, particularly beetroot, contributes to significant agricultural waste. In 2021, sugar beet farming yielded 269.19 million tons from 4.5 million hectares globally, according to FAO estimates. Beetroot processing, along with other food industries, generates approximately 1.3 billion tons of by-products such as peels and pomaces each year [12]. In addition, global apple production ranges from 82 to 87 million tons per year [13], further contributing to the substantial volume of agro-food waste [14]. The by-products obtained from potato, apple, and beetroot waste, frequently discarded in food processing operations, are abundant sources of bioactive compounds, including phenolic compounds and carotenoids [15,16,17]. Phenolic compounds, in particular, are well-documented for their potent antioxidant properties, which enable them to neutralize reactive oxygen species and reduce oxidative stress [18]. They are categorized as secondary metabolites, playing a critical role in plant defense mechanisms and human or animal health by reducing the risks associated with oxidative stress [19]. Recent studies suggest that phenolic compounds present in larval-rearing substrates can influence the accumulation of these bioactive compounds in BSFL [20]. When the phenols from red potato, apple, and beetroot peels are effectively transferred to the larvae, the resulting biomass could serve as an enriched protein source with functional antioxidant properties, providing benefits for both nutrition and health [4]. However, to fully assess the potential of enriched larval biomass for use in animal feed, a comprehensive analysis of both its benefits and risks is essential.

In this context, the aim of the present study is to valorize agricultural by-products, specifically apple peels (*Malus domestica*), potatoes (*Solanum tuberosum*), and red beetroot peel (*Beta vulgaris*), as rearing substrates for BSFL, with the scope to obtain a biomass product enriched with phenolic compounds. The study was validated through the comprehensive characterization of the biomass, including proximate analysis, the quantification of the total phenolic and total flavonoid contents, and antioxidant activity essays (e.g., CUPRAC, ABTS, and DPPH). Furthermore, in terms of processing, rheological profiling was conducted to identify the product’s technological qualities and its suitability for future final products. To address potential safety concerns for consumers, the mycotoxicological risk associated with the biomass was also analyzed, given that agro-food waste can carry fungal and mycotoxin contamination. Overall, the present study aimed to characterize BSFL reared on phenolic-rich agro-industrial substrates and to evaluate the feasibility of utilizing agricultural by-products to obtain phenolic-enriched insect biomass under practical rearing conditions. Despite the inherent methodological constraints associated with pilot-scale experimentation, the study was designed to generate robust baseline data on the substrate-driven modulation of larval composition and functional properties.

## 2. Materials and Methods

### 2.1. Purchasing By-Products Rich in Phenols and Flavonoids (Red Beetroot Peel, Red Potato Peel, and Red Apple Peel)

Several substrate variants containing beetroot, apple and potato peel by-products were prepared to assess the potential of BSFL to degrade phenol-rich organic matter. These materials, beetroot peel by-products (BBPs), apple peel by-products (ABPs) and potato peel by-products (PBPs) were selected due to their phenolic compound content and their large-scale availability as food waste [9,15,17]. The substrate ingredients were sourced from a local grocery store. After purchasing, the collected products were thoroughly washed, peeled, and manually processed. The by-products were cut into small flakes to increase their surface area, thus promoting larval consumption.

#### BSFL Rearing and Experimental Design

To conduct the experiment, neonate larvae of *H. illucens* were obtained from Nasekomo EAD in Lozen, Bulgaria. A total of nearly 100,000 first-instar larvae, averaging 0.5 mm in size, were purchased and transported under controlled conditions. The experimental design included separate containers for each feeding model, as shown in Table 1. All the containers were provided with standardized chicken feed as the main substrate and primary nutritional source (Pro Mix-ProntoVet Promark Trade, Bogata, Mureș County, Romania). To assess the impact of phenolic compounds, subsequent treatments were prepared by adding the substrate with 2%, 10%, and 20% of beetroot peels, potato peels, and apple peels, resulting in nine variants. Each container was supplied with a single type of peel at the designated concentration. The control group comprised larvae reared exclusively on a standard chicken feed diet. To ensure reproducibility, each experimental condition was performed in duplicate. Rearing containers of varying sizes were used in this study, which may have influenced larval density. As larval density can affect substrate consumption and the nutritional composition of BSFL, particularly crude fat content [21], this represents a potential confounding factor that should be considered when interpreting the substrate-specific effects.

The endpoint of the experiment was determined by the percentage of larvae reaching the prepupal stage, characterized by darkened exoskeletons and increased rigidity. The slaughtering moment was defined as the point at which over 20% of the larvae in a given model exhibited these characteristics. Since BSFL undergo asynchronous development, variations in slaughtering times were observed among experimental groups and are debated for each case in Section 4. Throughout the experiment, the environmental conditions were maintained at room temperature (23 ± 2 °C), without additional control of humidity. Each container was covered with a fine mosquito mesh to ensure proper aeration while preventing larval escape. The total experimental duration was 32 days, during which larval growth, survival rate, and development time were monitored. Before conducting any further analyses, the harvested larvae were subjected to freeze drying to ensure the preservation of their biochemical composition.

### 2.2. Proximate Composition

The BSFL were freeze dried until a constant weight (approximately 72 h), then ground into a fine powder and sieved to ensure uniform particle size (the mesh number was ten; powders had particle sizes < 2 mm) for analysis. The ground samples were subjected to proximate composition analysis using methods established by the Association of Official Analytical Chemists [22]. The dry matter content was measured after the samples reached a constant weight (oven drying at 105 °C, method 934.01; [22]). The ash content was determined by incinerating samples in a muffle furnace at 550–600 °C (method 942.05) until only inorganic residue remained [23]. The total fat was extracted using the Soxhlet method with hexane as solvent (method 920.39) [24]. The crude protein was analyzed using the Kjeldahl method, which involves digestion, neutralization, distillation, and titration to determine total nitrogen content (method 984.13). A nitrogen-to-protein conversion factor of 6.25 was used [25]. All analyses were conducted in duplicate (*n* = 2), and the results were expressed as the mean ± standard deviation.

### 2.3. Colorimetric Assay

We assessed each substrate’s color properties using a portable spectrophotometer (DS-700D; CHNSpec Technology (Zhejiang) Co., Ltd., Hangzhou, China). Following the methodology outlined by Cao et al. (2023) and Teleky et al. (2025), we recorded the L* (lightness), a* (redness/greenness), and b* (yellowness/blueness) parameters from three randomly selected areas [26,27].

### 2.4. Rheologic Assay

Freeze-dried BSFL powder from different rearing substrates were reconstituted in distilled water to 15% (*w*/*w*) protein concentration. The samples were stored at 4 °C overnight to ensure complete hydration. Rheological analyses were performed using a modular compact rheometer (Anton Paar MCR 72, Anton Paar, Graz, Austria) equipped with a Peltier-controlled plate-to-plate system (P-PTD 200/Air, (Anton Paar, Graz, Austria) for accurate temperature regulation within the range of 5–150 °C. Approximately 3 mL of each sample was placed between the plates, with the upper plate consisting of a smooth, parallel geometry (50 mm diameter) and the lower plate maintained at 25 °C, separated by a 1 mm gap [28]. Any excess material was carefully removed prior to analysis, and the samples were equilibrated for 5 min to achieve thermal stability. The measurements were conducted in duplicate while the shear rate was progressively increased from 5 to 300 s^−1^.

### 2.5. Ultrasound-Assisted Extraction

For the in vitro assessments regarding the total phenolic content, the total flavonoid content and the antioxidant potential of the samples, methanolic extracts were prepared. Each sample (1 g of BSFL powder) was mixed with 10 mL of methanol and homogenized using an orbital shaker for 10 min. The homogenized mixture was placed into an ultrasonic bath (Elma Schmidbauer GmbH, Singen, Germany) for 30 min at room temperature to enhance the extraction efficiency, followed by centrifugation for 10 min at 9000 rpm to separate the solid residues from the liquid phase. The supernatant was collected and filtered using 40 µm injection filter to remove any remaining particulate matter. The extracts were used to determine both total phenolic and total flavonoid contents and also to investigate the antioxidant capacity of the samples.

### 2.6. Total Flavonoids Content (TFC)

To determine the TFC in BSFL samples reared on agro-food by-product-added substrates, the methanolic extracts were subjected to the spectrophotometric method described by Ștefănescu et al. 2024 [29] as follows. The extracts (300 µL) were diluted with 720 µL of distilled water, followed by the addition of 90 µL of 5% NaNO_2_. After a 5 min of incubation (in the dark), 90 µL of 10% AlCl_3_ was added, and the mixture was incubated for another 5 min. Next, 600 µL of 1 N NaOH was introduced. The absorbance was measured at 510 nm, using quercetin as the reference standard. All the determinations were performed in triplicate, and the TFC was reported as milligram quercetin equivalent per gram of sample (mg QE/g sample).

### 2.7. Total Phenolic Content

The phenolic content was determined using the Folin–Ciocalteu method [30] with a few adjustments for microplate analysis. First, 25 µL aliquots of each extract were combined with 1.8 mL of distilled water in a 24-well microplate. Next, 125 µL of 0.2 N Folin–Ciocalteu reagent was added, and the mixture rested at room temperature for 5 min. Subsequently, 340 µL of a 7.5% (*w*/*v*) Na_2_CO_3_ solution was added, creating the optimal initial pH (around ten) for the redox reaction between the phenolic compounds and the Folin–Ciocalteu reagent. Afterward, the solutions incubated in the dark at 25 °C for 2 h. The blank was prepared with methanol, and absorbance was measured at 760 nm using a BioTek Instruments microplate reader (BioTek Instruments- Agilent Technologies, Winooski, VT, USA). All measurements were performed in triplicate. A standard curve was generated with gallic acid (0.01–1.00 mg/mL), and the TPC of the samples was expressed as milligram gallic acid equivalent per gram sample (mg GAE/g of sample).

### 2.8. Antioxidant Capacity Assays

Antioxidant capacity was determined using three different methods described in the following.

#### 2.8.1. Cupric Ion Reducing Antioxidant Capacity (CUPRAC)

For this determination, the method described by Apak et al. 2004 was used [31]. The CUPRAC reagent was freshly prepared by mixing an ammonium acetate buffer solution (1 M, pH 6.8–7.0), 10 mM CuCl_2_, and 7.5 mM neocuproine (in ethanol) in a 1:1:0.5 ratio. The samples (25 µL), blanks, or Trolox standards (10–1000 µM) were added to 96-well plates, in triplicate (*n* = 3), followed by 175 µL of CUPRAC reagent. The plates were incubated in the dark, at room temperature, for 30 min. The absorbance was read at 450 nm, and the total antioxidant capacity (TAC) was calculated using the Trolox calibration curve.

#### 2.8.2. DPPH Assay

The antioxidant activity of the samples was determined also using the 1,1-diphenyl-2-picrylhydrazyl (DPPH) free radical scavenging capacity technique, developed by Brand-Williams et al. [32]. To determine the antioxidant value of the samples, they were prepared in triplicate (*n* = 3); 35 μL of previously extracted aqueous samples was mixed with 250 μL of methanolic DPPH solution. The reaction solution was incubated for 30 min at room temperature, in the dark, before measuring the absorbance at 515 nm using a multi-mode plate reader (BioTek, Winooski, VT, USA). The results were calculated using the following formula: Inhibition (%) = ((Abs blank − Abs sample)/Abs blank) × 100, and were expressed as μM Trolox equivalents/100 g sample [32].

#### 2.8.3. ABTS Assay

Furthermore, the radical scavenging capacity of the samples against the 2,2′-azino-bis 3-ethylbenzothiazoline-6-sulfonic acid (ABTS) radical anion was determined according to the procedure described by Arnao et al. [33] with a modification to adapt it to 96-well microplates [34]. Twenty microliters (20 μL) of Trolox at different concentrations or BSG (bovine serum gamma globulin) extracts were added, in triplicate (*n* = 3), to 170 μL of ABTS●^+^ solution. The reaction mixture was incubated for 6 min in the dark at room temperature. Subsequently, absorbance was measured at 734 nm using a microplate reader. The results were expressed in μmol Trolox eqivalent/100 g sample.

### 2.9. Mycotoxin Analysis

To assess the safety of BSFL reared on phenol-rich substrates, several mycotoxicological analyses were performed. The study targeted two major mycotoxins, Aflatoxin B1 (AFB1) and Zearalenone (ZEA), using commercial ELISA kits from R-Biopharm AG. For Aflatoxin B1, detection was conducted using the RIDA^®^QUICK FAST Aflatoxin B1 kit (the limit of detection was 2 µg/kg). Ground larvae samples were extracted with solvent (methanol), filtered, and analyzed per kit instructions. The absorbance was measured at 450 nm, and the AFB1 concentrations were calculated from a standard curve. For Zearalenone, the RIDA^®^SCREEN FAST ZEA (with the limit of detection 17–41 μg/kg) kit was used. The samples were extracted with water, centrifuged, and diluted. The ELISA procedure followed the manufacturer’s protocol, with the absorbance read at 450 nm and ZEA levels determined via the calibration curve.

### 2.10. Statistical Analysis

All the experiments were conducted in duplicate or triplicate, as specified in the respective sections. The results are reported as the mean ± standard deviation (SD). Statistical analyses were performed using GraphPad Prism (version 9.3.0; GraphPad Software Inc., San Diego, CA, USA). Prior to the analysis of variance (ANOVA), data were assessed for normality using the Shapiro–Wilk test and for the homogeneity of variances using Levene’s test. When these assumptions were not met, the results were interpreted with caution in light of the limited number of biological replicates. Data were neither transformed nor analyzed using non-parametric tests, as such approaches were not expected to improve the reliability of the results given the experimental constraints. A two-way ANOVA, followed by Dunnett’s or Tukey’s multiple comparison tests, was used to evaluate the statistical differences between the control group and the various substrate treatments. Statistical significance was defined as *p* < 0.05.

## 3. Results

### 3.1. Characterization of Organic Substrates and Slaughtering Times

Due to the asynchronous development of BSFL, variations in slaughtering times were noted among groups, as is detailed in the following. The first populations to reach the prepupal stage were those reared on PBP_20%_ and BBP_2%_, 20 days after the experiment’s initiation. The second group, consisting of larvae reared on BBP_10%_ reached the prepupal stage at 22 days. The third group included larvae from the control group (chicken feed only), BBP_20%_, and all achieved the prepupal stage by day 23. The fourth group, fed ABP_20%_, reached the prepupal stage at 24 days post start. The final group consisted of the remaining populations, which reached the prepupal stage at day 32. This group included larvae reared on a range of substrates: PBP_2%_, PBP_20%_ (duplicate), PBP_10%_, PBP_10%_ (duplicate), ABP_20%_ (duplicate), ABP_2%_, PBP_2%_ (duplicate), ABP_2%_ (duplicate), ABP_10%_, ABP_10%_ (duplicate), and PBP_10%_ (duplicate). Interestingly, despite standardized rearing conditions (temperature, humidity, and substrate handling), developmental discrepancies were observed (Table 2).

### 3.2. Proximal Composition

In the subsequent analysis, the nutritional composition of the experimental groups of BSFL was characterized. This evaluation aimed to assess the influence of these specific agro-industrial by-products on the larvae’s nutritional profile, including moisture, protein, fat, and ash, with the results showed in Table 3.

Variability was observed among the experimental groups. The ash content showed a wide range of values, with PBP_2%_ exhibiting the highest level (24.94%), suggesting a strong mineral contribution from low-level potato peel inclusion. In contrast, ABP_20%_ (12.51%) and the control (12.71%) recorded the lowest ash contents. Protein content increased progressively with higher inclusion levels in both the ABP and BBP groups, reaching maximum values at 20% inclusion, 40.05% in ABP_20%_ and 40.09% in BBP_20%_. In contrast, PBP_20%_ exhibited the lowest protein content (30.89. Moisture content also varied considerably among groups: BBP_2%_ showed the highest moisture level (18.92%), while BBP_20%_ recorded the lowest (6.66%), indicating a strong moisture-reducing effect of higher beetroot peel inclusion. Fat content displayed a different pattern, with ABP_10%_ (18.04%) and PBP_2%_ (18.20%) achieving the highest lipid levels; meanwhile, PBP_20%_ had the lowest fat content (6.70%), demonstrating that increasing the potato peel concentration may reduce lipid deposition. Nitrogen-free extract (NFE), also known as the total carbohydrate content, was the highest in BBP_10%_ (30.34%), suggesting that moderate beetroot supplementation enhances carbohydrate accumulation. The lowest NFE values occurred in PBP_2%_ (13.91%) and ABP_10%_ (13.56%), reflecting limited carbohydrate content in these groups.

### 3.3. Colorimetric Assay

Colorimetric measurements revealed clear differences among larvae reared on phenolic-rich by-product substrates. Lightness (L*-100 indicates white and 0 indicates black) was highest in the PBP_2%_ group; meanwhile, the lowest values occurred in the control and ABP_20%_, showing that higher apple peel inclusion darkened the larvae.

In general, 2% inclusion produced lighter larvae, while 20% tended to reduce lightness. For the a* coordinate, the greatest redness was observed in PBP_10%_ and ABP_20%_, while the lowest value occurred in ABP_2%_. Both the apple and potato peel substrates increased redness at higher inclusion levels, whereas beetroot peel produced moderate, stable a* values. Yellowness (b*) peaked in PBP_2%_ and ABP_10%_, and was lowest in PBP_20%_ and BBP_2%_. High inclusion levels (20%) in all substrate types tended to decrease yellowness. The overall color difference (ΔE) was greatest in PBP_2%_ (*p* < 0.05), followed by BBP_10%_ and ABP_10%_, showing that potato peel substrates, especially at lower inclusion rates, produced the most pronounced color changes. The smallest ΔE occurred in ABP_20%_, indicating minimal deviation from the control. Collectively, both the substrate type and inclusion level markedly influenced larval pigmentation, with potato peel exerting the strongest effect (Table 4).

### 3.4. Rheological Assessment

**Figure 1:**
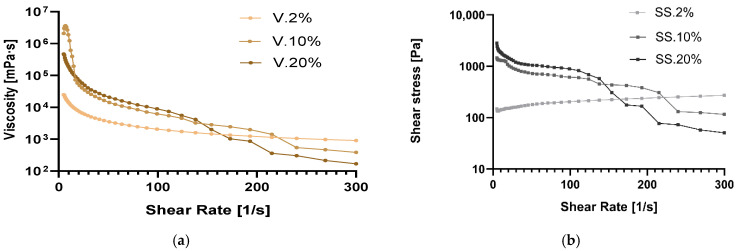

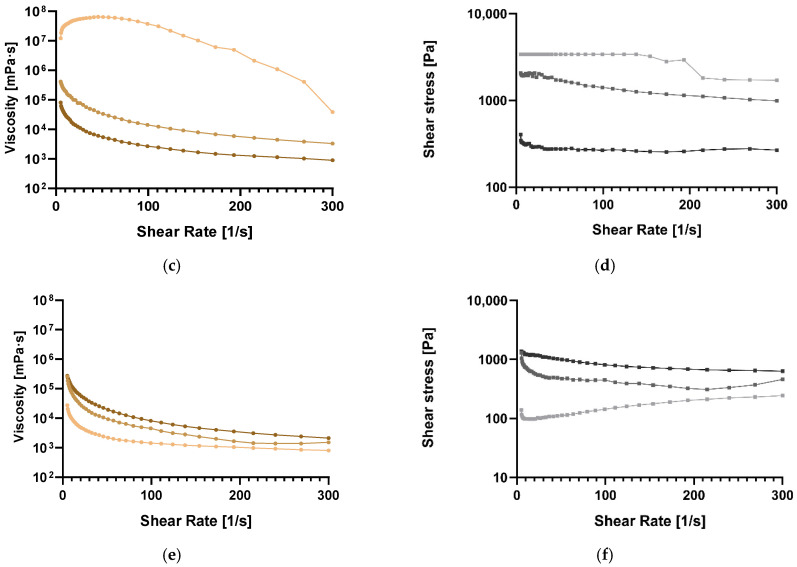
Viscosity and shear stress as a function of shear rate for the (**a**,**b**) ABP, (**c**,**d**) BBP, and (**e**,**f**) PBP.

The rheological assessment of the reconstituted BSFL protein suspensions revealed that all the experimental formulations exhibited pronounced non-Newtonian, shear-thinning behavior (Figure 1). This behavior is similar to the viscoelastic characteristics typically reported for insect protein dispersions and other high-protein food matrices [35,36]. Across all samples, viscosity markedly decreased with increasing shear rate (5–300 s^−1^), indicating that applied shear disrupted the internal associative structures governing fluid resistance [28].

The average apparent viscosity ranged from 5.5 × 10^3^ mPa·s (PBP_2%_) to 3.6 × 10^7^ mPa·s (BBP_2%_), highlighting how the inclusion of phenolic-rich by-products considerably influenced the fluid’s structural organization. At the lowest shear rate, BBP_2%_ exhibited a high initial viscosity of 1.2 × 10^7^ mPa·s, which decreased to 3.9 × 10^4^ mPa·s at 300 s^−1^. This reduction indicates important intermolecular networking, most probably resulting from protein–polyphenol crosslinking facilitated by beetroot-derived betacyanins and phenolic acids [37]. These compounds can promote the formation of covalent or non-covalent complexes with amino acid side chains, thus enhancing structural rigidity while also making the structure sensitive to shear disruption [38].

ABP_10%_ and ABP_20%_ exhibited high initial viscosities of 2.1 × 10^6^ and 5.7 × 10^5^ mPa·s, respectively, but broke down significantly under shear rate, ending up below 400 mPa·s. This high viscosity suggests that the phenolics from apple peel formed a moderately strong but shear-sensitive network with larval proteins. Conversely, PBP-based groups, particularly PBP_2%_ and PBP_10%,_ had lower viscosities (2.7 × 10^4^ to 2.6 × 10^5^ mPa·s at 5 s^−1^) and displayed minimal changes under shear, indicating that starch–protein [39] and pectin–protein [40] interactions were important. While polysaccharide entanglement offered some viscosity resistance, it lacked the strong bonding seen in phenolic–protein networks [37].

### 3.5. Mycotoxin Analysis

The results of this essay are presented in Table 5. Aflatoxin B1 (AFB1) concentrations were generally low and showed only minor differences among treatments. The highest AFB1 concentration was observed in the control group, while the lowest value occurred in the BBP_20%_ treatment. Experimental groups with apple peel (ABP), beetroot peel (BBP) and potato peel (PBP) additions produced AFB1 values ranging between 2.26 and 2.59 µg/kg. Overall, AFB1 varied little between substrate types or inclusion rates and remained comparable to the control.

Zearalenone (ZEA) concentrations varied substantially among the groups. The highest ZEA level was recorded for PBP_2%_, closely followed by BBP_10%_. The lowest ZEA value was observed in PBP_10%_; the control showed a similarly low value. Apple peel treatments produced intermediate ZEA levels, whereas beetroot and potato peels showed both some of the highest and some of the lowest ZEA values depending on the inclusion rate. Notably, ZEA standard deviations were large in several groups (e.g., ABP_10%_, PBP_2%_, and PBP_20%_), indicating substantial sample variability.

AFB1 concentrations were low and fairly uniform across all substrates and inclusion levels, while ZEA concentrations were strongly affected by both the substrate type and inclusion rate.

### 3.6. Total Flavonoid Content (TFC)

**Figure 2:**
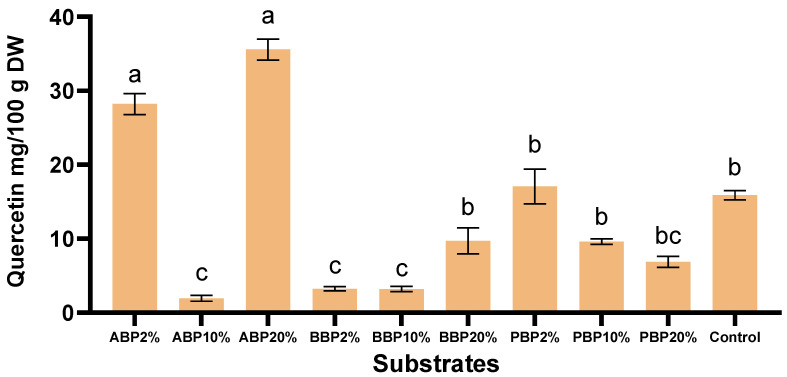
Total flavonoid content (TFC) of BSFL reared on phenolic-rich agro-industrial substrates. Values are expressed as mean ± SD (*n* = 3). Statistical analysis was performed using two-way ordinary ANOVA followed by Tukey’s multiple comparison test. Different letters above the bars indicate statistically significant differences among treatments (*p* < 0.05); treatments sharing at least one letter are not significantly different.

The flavonoid content of BSFL showed substantial variation across the different phenolic-enriched rearing substrates (Figure 2). Among all the experimental groups, the highest TFC value was obtained for ABP_20%_ (35.578 ± 1.41 Quercetin mg/100 g DW), indicating that high-level supplementation with apple peels resulted in the strongest flavonoid accumulation. This was followed by ABP_2%_, which also produced markedly elevated flavonoid levels compared to the control. In contrast, the lowest TFC (*p* < 0.05) was recorded in the ABP_10%_ group (1.95 ± 0.40 Quercetin mg/100 g DW), representing a sharp reduction relative to both the lower and higher apple peel inclusion levels.

BBP resulted in comparatively low flavonoid contents across all inclusion levels. TFC ranged from 3.23 Quercetin mg/100 g DW at 2% to 9.70 Quercetin mg/100 g DW at 20%, with all BBP values substantially lower than both the control and most ABP treatments. Although BBP_20%_ produced the highest value within this substrate group, it remained far below the levels observed in the high- and low-level apple peel treatments.

The potato peel treatments showed intermediate flavonoid contents relative to the other substrate types. The highest value in this group was recorded at 2% inclusion (17.07 ± 2.35 Quercetin mg/100 g DW), slightly above the control. However, TFC declined at higher levels of supplementation, decreasing at 10% and further at 20%.

When compared with the control, only ABP_2%_, ABP_20%_, and PBP_2%_ resulted in notably elevated flavonoid contents (*p* < 0.05), while all other treatments—particularly BBP and most PBP groups—expressed lower TFC values. Overall, the findings indicate that apple peel supplementation, especially at 20%, was most effective in enhancing flavonoid content in the larvae, whereas beetroot peel supplementation had the weakest effect. Potato peel supplementation produced moderate increases only at low inclusion levels.

### 3.7. Total Phenol Content (TPC)

**Figure 3:**
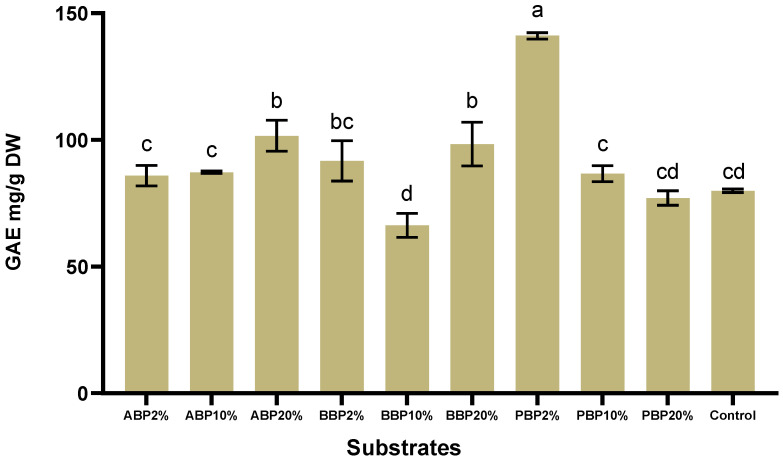
Total phenolic content (TPC) of BSFL reared on phenolic-rich agro-industrial substrates. Values are expressed as mean ± SD (*n* = 3). Statistical analysis was performed using two-way ordinary ANOVA followed by Tukey’s multiple comparison test. Different letters above the bars indicate statistically significant differences among treatments (*p* < 0.05); treatments sharing at least one letter are not significantly different.

The phenolic content of BSFL, shown in Figure 3, varied considerably depending on the type and inclusion level of the phenolic-rich plant by-products incorporated into the rearing substrate. The highest TPC value was recorded in the PBP_2%_ group (141.046 ± 1.269 GAE mg/g DW), indicating a strong phenolic accumulation in larvae fed with low-level potato peel supplementation. In contrast, the lowest TPC was observed in the BBP_10%_ group (66.2635 ± 4.773 GAE mg/g DW), representing the weakest phenolic enrichment effect among all experimental treatments.

TPC increased progressively with the level of supplementation within the ABP group, ranging from 85.84 GAE mg/g DW at 2% to 101.61 GAE mg/g DW at 20%, with the highest value observed at the 20% inclusion level. BBP showed a non-linear pattern: while BBP_2%_ exhibited a moderately high TPC (91.66 ± 7.93 GAE mg/g DW), BBP_10%_ decreased markedly (*p* < 0.05), and BBP_20%_ increased again to 98.28, approaching the values of the ABP_20%_ group. The potato peel treatments displayed a distinct trend compared to the other substrates. TPC peaked sharply at 2% inclusion, showing the single highest value among all treatments, but decreased substantially at higher inclusion levels, falling to 86.64 GAE mg/g DW at 10% and 77.003 GAE mg/g DW at 20%.

When compared to the control group (79.83 ± 0.70), several treatments showed markedly elevated TPC, most notably PBP_2%,_ ABP_20%_, BBP_20%,_ and BBP_2%_. In contrast, BBP_10%,_ PBP_20%_, and ABP_2%_ expressed phenolic contents close to or below the control. Overall, potato peel supplementation at low inclusion levels produced the strongest enhancement of phenolic accumulation, whereas higher levels tended to reduce TPC. Apple peel supplementation resulted in a consistent dose-dependent increase, while beetroot peel substrates led to more variable outcomes depending on inclusion level.

### 3.8. Antioxidant Capacity Assays

The antioxidant capacity of BSFL was determined using three different methods: CUPRAC, DPPH, and ABTS.

#### 3.8.1. CUPRAC (Cupric Ion Reducing Antioxidant Capacity)

**Figure 4:**
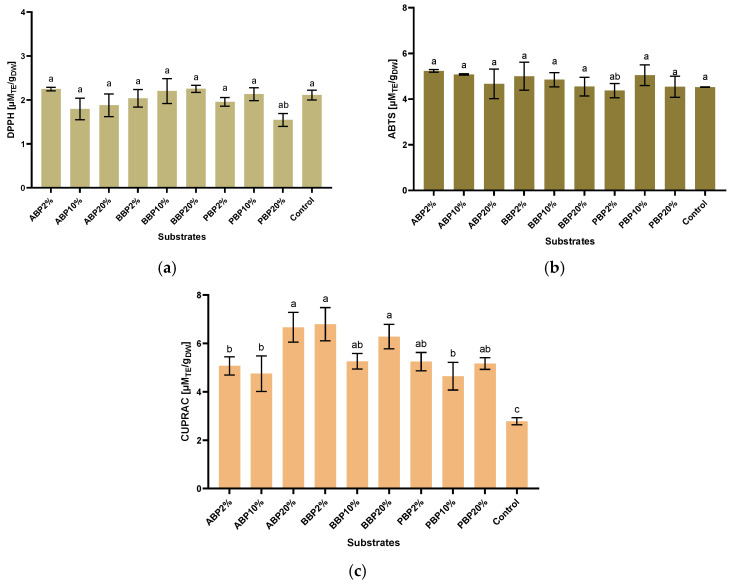
Antioxidant capacity of BSFL reared on agro-industrial substrates, assessed by (**a**) DPPH, (**b**) ABTS, and (**c**) CUPRAC assays. The results are expressed as the mean ± SD (*n* = 3). Statistical analysis was performed using two-way ANOVA followed by Tukey’s multiple comparison test. Different letters above bars indicate statistically significant differences among treatments (*p* < 0.05); treatments sharing at least one letter are not significantly different.

The CUPRAC essay revealed clear differences in antioxidant capacity among larvae reared on substrates enriched with phenolic-rich by-products. The highest CUPRAC value was observed in the BBP_2%_ group (6.79 ± 0.68 µM_TE_/g_DW_), followed closely by ABP_20%_ (6.66 ± 0.61) and BBP_20%_ (6.27 ± 0.50 µM_TE_/g_DW_). These results indicate that beetroot peel substrates, particularly at low inclusion levels, produced the strongest enhancement in larval antioxidant capacity. In contrast, the lowest CUPRAC value among all groups was recorded in the control (2.78 ± 0.15 µM_TE_/g_DW_), confirming the influence of phenolic-enriched diets on antioxidant potential (*p* < 0.05). Within the apple peel treatments, the antioxidant capacity increased with higher inclusion levels, rising from 5.07 ± 0.37 µM_TE_/g_DW_ (ABP_2%_) to 6.66 ± 0.61 µM_TE_/g_DW_ (ABP_20%_). The intermediate group ABP_10%_ (4.74 ± 0.73 µM_TE_/g_DW_) showed greater variability and a modest antioxidant response compared to the 2% and 20% levels. Beetroot peel groups showed consistently high values, with BBP_10%_ (5.26 ± 0.31 µM_TE_/g_DW_) also outperforming the control and remaining similar to other enriched substrates. Potato peel treatments produced moderate CUPRAC values (PBP_2%_ 5.24 ± 0.38 µM_TE_/g_DW_; PBP_20%_ 5.16 ± 0.24 µM_TE_/g_DW_), with PBP_10%_ displaying the lowest antioxidant response within this substrate category (4.64 ± 0.57 µM_TE_/g_DW_) (Figure 4).

#### 3.8.2. DPPH (1,1-Diphenyl-2-picrylhydrazyl)

The DPPH radical scavenging activity of BSFL varied across the different phenolic-enriched rearing substrates. Among all treatments, BBP_20%_ (2.25 ± 0.07 µM_TE_/g_DW_) exhibited the highest antioxidant activity, closely followed by ABP_2%_ (2.24 ± 0.04 µM_TE_/g_DW_) and BBP_10%_ (2.20 ± 0.28 µM_TE_/g_DW_). The lowest value was recorded for PBP_20%_ (1.54 ± 0.14 µM_TE_/g_DW_), representing the weakest radical scavenging capacity among the enriched diets.

Within ABP, antioxidant activity remained relatively consistent, ranging from 1.79 to 2.24 µM_TE_/g_DW_, with the highest value at 2% inclusion. BBP showed a clear upward trend; increasing inclusion levels tended to improve DPPH scavenging activity, culminating in the highest overall value at 20% supplementation. In contrast, within PBP, the highest activity was observed at 10% inclusion (2.13 ± 0.14 µM_TE_/g_DW_), whereas 20% supplementation markedly reduced activity to the lowest level observed across all groups (Figure 4). When compared with the control group (2.11 ± 0.11 µM_TE_/g_DW_), several treatments demonstrated higher antioxidant capacity, most notably BBP_20%,_ ABP_2%,_ BBP_10%,_ BBP_2%,_ PBP_10%,_ and ABP_20%_. In contrast, ABP_10%,_ PBP_2%,_ and particularly PBP_20%_ exhibited lower activity than the control.

#### 3.8.3. ABTS Stands for 2,2′-Azino-bis(3-ethylbenzothiazoline-6-sulfonic Acid)

The ABTS radical scavenging activity of BSFL differed among the phenolic-enriched rearing substrates. The highest ABTS value was obtained from ABP_2%_ (5.23 ± 0.05 µM_TE_/g_DW_), indicating that low-level apple peel supplementation produced the greatest enhancement in antioxidant capacity. This was closely followed by ABP_10%_ (5.0735 ± 0.030 µM_TE_/g_DW_) and PBP_10%_ (5.04 ± 0.45 µM_TE_/g_DW_). In contrast, the lowest value was recorded for the control group (4.52 ± 0.01 µM_TE_/g_DW_), with PBP_20%_ (4.53 ± 1.46 µM_TE_/g_DW_) and BBP_20%_ (4.54 ± 1.04 µM_TE_/g_DW_) showing similarly low antioxidant activity.

Regarding ABP, antioxidant activity decreased progressively with increasing supplementation level, declining from 5.23 µM_TE_/g_DW_ at 2% to 4.66 µM_TE_/g_DW_ at 20%. In BBP, a comparable decreasing pattern was observed, with ABTS values dropping from 5.00 µM_TE_/g_DW_ at 2% to the lowest BBP value at 20% inclusion. The PBP showed a different trend: antioxidant activity peaked at 10% supplementation (5.04 ± 0.45 µM_TE_/g_DW_) but declined at both lower (4.36 ± 1.30 µM_TE_/g_DW_) and higher (4.53 ± 1.46) inclusion levels.

When compared with the control group, nearly all the enriched diets resulted in higher ABTS radical scavenging activity, with the exception of PBP_2%,_ PBP_20%,_ and BBP_20%,_ which clustered close to the control value. The apple peel treatments produced the strongest overall responses among the three substrate types, especially at lower inclusion levels, whereas beetroot and potato peel supplementation showed more variable and concentration-dependent effects.

## 4. Discussion

The present results demonstrate that phenolic-rich substrates can substantially alter the nutritional, colorimetric, and antioxidant characteristics of BSFL, emphasizing their potential to enhance insect-derived ingredients within sustainable food and feed systems [1].

All BSFL protein suspensions exhibited clear non-Newtonian, shear-thinning behavior, consistent with findings for other insect and high-protein systems [35,36]. Viscosity decreased sharply with increasing shear rate, indicating the disruption of associative structures within the protein matrix. The wide range of apparent viscosities across treatments demonstrates that phenolic-rich by-products substantially modified the structural organization of the suspensions [41]. These results align with previous observations that phenolic-rich diets increase viscosity and alter functional behavior in BSFL-derived materials. Navajas-Porras et al. (2024) found increased thickness and flow resistance in BSFL fed on coffee and blood meal due to the higher phenolic content and protein changes [20]. Research on BSFL predominantly focuses on their nutritional aspects, such as protein, fat, and amino acid content, as noted by Lu et al. (2022) [42]. In contrast, our study adopts a novel perspective by examining the effects of agro-industrial substrates high in phenolic compounds on BSFL. We discovered that these substrates alter the rheological properties of BSFL biomass. Notably, we observed a marked increase in thickness at low shear rates and enhanced shear-thinning behavior. These modifications not only contribute to the physical properties of the biomass but also improve its functional quality, extending beyond mere nutritional enhancements.

Flavonoid accumulation in BSFL varied markedly in response to the phenolic-enriched substrates, suggesting substrate-specific metabolic interactions. Apple peel supplementation produced the most pronounced effects, with ABP_20%_ yielding the highest TFC, followed by ABP_2%_. This non-linear pattern, where intermediate inclusion (ABP_10%_) resulted in a sharp reduction, may indicate an inhibitory mechanism affecting flavonoid uptake or conversion at mid-level concentrations. Although the literature highlights the negative effects of phenolic compounds on unadapted insects [43], the ability of larvae to deposit flavonoids in their biomass remains unclear. In contrast, beetroot peel consistently produced low TFC values across all inclusion levels. While the flavonoids present in beetroot, such as quercetin, kaempferol, and rutin [44,45], can accumulate in larval biomass, they simultaneously exert a strong inhibitory effect on larval metabolism through their enzyme-suppressing activity [46]. Potato peel generated intermediate responses, with elevated TFC only at 2% inclusion and declining values at higher levels, suggesting that excessive supplementation may reduce metabolic efficiency or induce feedback inhibition [43]. Overall, these results support the hypothesis that the flavonoid content of BSFL is strongly influenced by both the type and concentration of phenolic substrates, with apple peel particularly at high inclusion, promoting the greatest accumulation, and beetroot peel appearing to exert the weakest effect. The observed non-linear trends further suggest potential metabolic saturation or inhibitory processes that warrant deeper investigation.

The proximate composition of BSFL was markedly influenced by both the type and inclusion level of the phenolic-rich substrates. The pronounced variation in ash content among treatments suggests differential mineral deposition associated with substrate composition. The highest ash level observed in the PBP_2%_ group indicates that low concentrations of potato peel provided a mineral profile readily assimilated by the larvae. In contrast, ABP20_%_ and the control group exhibited the lowest ash values, meaning either reduced mineral availability or decreased mineral retention at higher apple peel inclusion levels [47]. Although these factors may not fully explain the observed extremes, inherent differences in mineral composition between by-products, particularly the higher mineral content of potato peels [48] compared with apple peels [49], likely contributed to this trend.

Protein content showed clear increasing trends in the ABP and BBP treatments, reaching the highest values at 20% inclusion. This pattern indicates that larval protein deposition was primarily substrate-dependent and influenced by frass composition rather than direct metabolic effects alone. Phenolic-rich apple and beetroot peels at higher inclusion levels may therefore have improved substrates’ nutritional quality, indirectly enhancing protein accumulation. Consistent with this substrate-dependent effect, previous comparisons of rearing substrates, including chicken feed, blood meal, spent coffee grounds, and mixtures of blood meal with spent coffee grounds, reported significant differences in larval protein and fatty acid profiles, accompanied by variations in antioxidant capacity, with the highest levels observed in larvae reared on blood meal [20]. Conversely, the reduced protein content in PBP_20%_ suggests that excessive potato peel inclusion may dilute available nitrogen or impair digestive efficiency, thereby limiting protein deposition, although in advanced mammals, including humans, potato consumption has been associated with enhanced protein synthesis rates [50].

Moisture content was likewise affected by substrate composition. The markedly higher moisture level in BBP_2%_ compared with BBP_20%_ indicates that increasing beetroot peel inclusion may reduce water retention in larval biomass. This effect may be linked to the fibrous nature of beetroot peels [51], which can influence water balance [41] and potentially modify feeding dynamics at higher inclusion levels. Fat content exhibited substrate-specific responses. The elevated lipid levels in ABP_10%_ and PBP_2%_ suggest that moderate apple peel and low potato peel inclusion [52] may promote lipid deposition [53], possibly through improved energy availability [54] or balanced nutrient supply. In contrast, the reduced fat content observed in PBP_20%_ indicates that excessive potato peel supplementation may limit energy-dense components or redirect metabolism away from lipid storage.

However, as BSFL undergo substantial metabolic shifts during late larval development—particularly affecting feeding behavior and lipid metabolism [55]—part of the variation in nutritional composition may reflect their developmental stage rather than substrate effects alone. Differences in growth rate and feeding intensity among experimental groups may have influenced lipid accumulation and overall nutrient allocation. Accordingly, a prolonged developmental time in certain treatments may have induced metabolic adjustments or starvation-related responses, contributing to reduced lipid deposition. Given that nutrient allocation in BSFL is closely linked to developmental progression, some of the observed compositional differences may therefore arise from developmental asynchrony and associated metabolic constraints, representing a confounding factor as well as a limitation of this study that warrants the cautious interpretation of substrate-specific effects.

Colorimetric measurements indicate that both the substrate type and inclusion level influenced larval pigmentation, likely through interactions between dietary phenolics and cuticle formation pathways. Lightness (L*) was highest in PBP_2%_ and lowest in the control and ABP_20%_ groups, suggesting that higher apple peel inclusion promoted cuticle darkening. This effect is consistent with the increased availability of the quinone-associated protein complex found in apples from oxidized phenols [56], which may also enhance melanization. In contrast, low-level potato peel supplementation may remain below the threshold required to trigger strong melanogenic responses, resulting in lighter larvae [57]. Redness (a*) increased at higher inclusion levels in both apple and potato peel treatments, which may reflect the accumulation of melanin intermediates derived from phenolic oxidation that could further enhance [58] its accumulation by larvae. Beetroot peel, despite its endogenous pigments, produced relatively stable a* values, likely due to the poor stability of betalains in the insect gut and limited transfer to the cuticle.

Yellowness (b*) generally declined at 20% inclusion across all substrates, suggesting that elevated phenolic intake shifts pigmentation toward darker melanin tones at the expense of yellow structural or carotenoid-associated hues. The largest overall color differences (ΔE) occurred in PBP_2%,_ BBP_10%,_ and ABP_10%_, indicating that phenolic-rich substrates can markedly alter larval coloration, with potato peel exerting the strongest effect. This may be linked to chlorogenic acid-mediated PPO activity, which is known to generate dark quinone pigments [56]. Even though all the experimental groups were processed under identical conditions, it is important to acknowledge that processing techniques may still influence colorimetric parameters [57]. Minor variations in handling, sample preparation, or equipment performance can introduce detectable differences in measured values. Nevertheless, the majority of the colorimetric data obtained in this study remained within the ranges reported in the literature [57], indicating that any processing-related effects were limited and did not compromise the overall reliability of the results.

Regarding mycotoxins, the AFB1 concentrations remained uniformly low across all treatments, with only minor variation among substrates and inclusion levels. The control group showed the highest concentration, whereas BBP_20%_ exhibited the lowest value. All by-product-enriched groups clustered within a narrow range (2.3–2.6 µg/kg), indicating that the inclusion of apple, beetroot, or potato peels did not markedly influence AFB1 accumulation. The AFB1 levels measured in our experimental groups complied with the European Commission Regulation (2006) [59], falling within the 1–15 µg/kg range. Notably, the European Union maintains some of the most stringent food contaminant standards globally [60]. Although previous reports described AFB1 levels in BSFL reared on contaminated substrates as remaining below the detection limit of 0.10 µg/kg [61], our findings detected concentrations between 2.3 and 2.6 µg/kg. In contrast, ZEA concentrations varied considerably among treatments, reflecting a stronger substrate-dependent effect. The highest ZEA levels occurred in PBP_2%_ and BBP_10%,_ whereas PBP_10%_ and the control exhibited the lowest values. Apple peel treatments produced intermediate concentrations, suggesting a moderate influence on ZEA accumulation. The concentrations measured in our experimental groups were within the regulatory limits established by both Chinese and European Commission food standards (50–350 µg/kg) [60], and all values remained below the maximum permitted threshold [62]. However, the highest concentration detected (256 µg/kg) exceeds the maximum limits established for several animal categories, including the following: piglets, gilts, puppies, kittens, and dogs and cats for reproduction (10 µg/kg); adult dogs and cats not intended for reproduction (20 µg/kg); sows and fattening pigs (25 µg/kg); and calves, dairy cattle, sheep, and goats (50 µg/kg). It also surpasses the limit set for cereals and cereal products other than maize by-products (200 µg/kg), while remaining below the maximum threshold established for maize by-products (300 µg/kg) [63]. These exceedances highlight the need for stringent safety monitoring to safeguard animal health. The pronounced variability observed, particularly in PBP_2%_, PBP_20%_, and ABP_10%_, suggests differences in larval biotransformation efficiency or metabolic responses to distinct phenolic profiles. Given the documented toxic effects of phenolic compounds on insects [64], such compounds may interfere with larval mycotoxin metabolism. Phenols derived from beetroot and potato peels may further modulate ZEA dynamics by altering gut redox conditions, affecting microbial ZEA transformation, or competing for detoxification enzymes [65]. Overall, while AFB1 remained consistently low and largely unaffected by substrate enrichment, ZEA levels were clearly substrate dependent, underscoring the divergent metabolic handling of these toxins by BSFL and the influence of phenolic-rich substrates on mycotoxin fate. Nevertheless, one limitation of the present study is the absence of a mycotoxin analysis of the raw rearing substrates. Consequently, it cannot be conclusively determined whether the detected mycotoxins resulted from bio-concentration during larval development or reflected residual contamination originating from the substrates themselves. This uncertainty represents an important limitation and should be addressed in future studies to better elucidate the dynamics of mycotoxin transformation and accumulation in BSFL rearing systems.

The phenolic content of BSFL differed markedly across treatments, confirming that both the type and inclusion level of phenolic-rich plant by-products strongly influenced larval phenolic accumulation. Among all groups, larvae reared on the 2% potato peel substrate exhibited the highest TPC, indicating that low-level supplementation is particularly effective in promoting phenolic enrichment. These findings align with the hypothesis that elevated phenolic exposure may affect larval metabolism and influence phenolic deposition within the biomass [65]. Conversely, the lowest TPC was observed in the 10% beetroot peel group, suggesting limited phenolic transfer at this inclusion level. A comparison with previously reported values indicates that the phenolic concentrations measured in this study generally exceed those documented in the literature [66]. Although differences in units required recalculation for methodological comparability, the adjusted values remained consistently higher than those in earlier reports. Substantial variability in phenolic content depending on rearing substrate has likewise been emphasized in previous studies [3]. Within the apple peel treatments, TPC increased progressively with higher supplementation, reaching its maximum at 20%, demonstrating a clear dose-dependent relationship. Despite concerns regarding the potential anti-nutritional effects of phenolic compounds, larvae reared on apple by-product substrates showed proportional increases in tissue phenolic deposition with increasing inclusion levels. This response may reflect physiological mechanisms such as enhanced sclerotisation, whereby phenolic compounds are incorporated into the cuticular matrix [67]. In contrast, potato peel supplementation exhibited a different pattern: although the 2% level produced the highest overall TPC, higher inclusion levels resulted in progressively lower values, suggesting the existence of a metabolic threshold beyond which phenolic accumulation becomes less efficient. A possible explanation involves the inhibitory effects of hydroxycinnamic acids present in potato-derived materials. While such effects have been described in the sweet potato weevil [68], similar mechanisms may operate in BSFL, potentially accounting for the reduced phenolic deposition observed at higher potato peel inclusion levels. These findings indicate that low-level potato peel supplementation exerts the strongest stimulatory effect on phenolic accumulation, whereas apple peel supports a more linear dose–response pattern and beetroot peel shows a non-linear inclusion-dependent effect.

Among the antioxidant assays, the CUPRAC method revealed clear differences in antioxidant capacity among larvae reared on phenolic-enriched substrates. The highest value was observed in BBP_2%_, followed by ABP_20%_ and BBP_20%_, indicating that beetroot peel at low inclusion levels effectively enhanced larval antioxidant potential. In contrast, the control group exhibited the lowest CUPRAC values, underscoring the contribution of phenolic-rich substrates to antioxidant capacity. These results suggest that, despite the potential inhibitory effects of phenolics on BSFL, controlled inclusion levels can enhance antioxidant properties of the biomass. Apple peel treatments showed a marked increase in antioxidant capacity with higher inclusion levels, while beetroot peel consistently produced elevated values. Potato peel supplementation generated more moderate responses and the lowest antioxidant capacity within this substrate category. Although the CUPRAC assay has not previously been applied to BSFL, studies in other insect species such as *Tenebrio molitor* support its suitability for evaluating endogenous antioxidant potential. Dietary enrichment with phenolic compounds has been shown to enhance larval antioxidant capacity; for example, Gulsunoglu-Konuskan et al. (2024) reported an increase from 20.50 to 28.86 mg TE/g larvae following supplementation with phenolic-rich substrates [69], a trend consistent with our observations. DPPH radical scavenging activity also varied substantially among treatments. The highest activity was recorded in BBP_20%_, followed by ABP_2%_ and BBP_10%_, whereas PBP_20%_ showed the weakest response. Beetroot peel exhibited a clear dose-dependent increase, likely related to its high betaine content and associated antioxidant properties [70]. Apple peel produced relatively stable responses, with the highest activity at 2% inclusion. In contrast, potato peel peaked at 10% but declined markedly at 20%, indicating concentration-sensitive effects. Overall, beetroot peel at higher inclusion levels elicited the strongest DPPH enhancement, while potato peel responses were more variable and apple peel effects were moderate but consistent. These patterns align with previous findings highlighting substrate-driven variability in BSFL antioxidant capacity. González et al. (2025) reported DPPH scavenging capacities ranging from 0.07 to 0.17 mg/mL [3]. Although differing units prevent direct quantitative comparison, both studies emphasize the central role of diet in modulating larval antioxidant profiles. The ABTS assay further confirmed substrate-dependent effects. The highest value was observed in ABP_2%_, supporting the hypothesis that low-level phenolic supplementation can enhance antioxidant capacity while minimizing potential inhibitory effects on non-adapted insect species [43]. The control group exhibited the lowest activity, with PBP_20%_ and BBP_20%_ showing similarly low values. Most enriched diets improved ABTS scavenging compared to the control, except PBP_2%_, PBP_20%_, and BBP_20%_, which remained comparable to baseline levels. The unexpectedly low ABTS activity in PBP_2%_ may be linked to specific phenolic constituents such as hydroxycinnamic acids and chlorogenic acid [71], although further investigation is required. Overall, apple peel supplementation (2–10%) was most effective in enhancing ABTS activity, whereas beetroot and potato peel treatments exhibited more variable, concentration-dependent responses. Similar variability has been reported by González et al. (2025), who observed ABTS values between 0.05 and 0.24 mg/mL [3]. Despite differences in units, both datasets highlight the decisive role of substrate composition in shaping antioxidant profiles. Collectively, these assays demonstrate that phenolic-rich by-products can enhance BSFL antioxidant capacity, although the magnitude and direction of the response depend strongly on substrate type and inclusion level. Apple peel at low concentrations and beetroot peel at higher concentrations appear to be the most effective, while potato peel produces moderate and less predictable effects. Nevertheless, the interpretation of these patterns should be approached with caution. A high degree of variability was observed among the experimental batches, influencing proximate composition, phenolic content, antioxidant activity, and colorimetric measurements. This variability likely reflects biological heterogeneity as well as subtle micro-environmental differences during rearing. Moreover, several responses, particularly antioxidant capacity and phenolic accumulation, displayed non-linear trends, suggesting possible metabolic thresholds or inhibitory effects. The inconsistent responses observed at higher inclusion levels (10% and 20%) further underscore the complex and substrate-dependent interactions governing larval metabolism, representing an important limitation of the present study.

A clear consideration of the experimental design is necessary when interpreting the present findings. The study was conceived to evaluate BSFL performance under semi-scaled rearing conditions using phenolic-rich agro-industrial substrates. Within this framework, rearing containers of varying sizes were employed, resulting in differences in total substrate mass among batches. Although the proportional inclusion of agro-industrial by-products was kept constant across treatments, the full standardization of container volume was not achieved. Consequently, the precise control of larval density was not possible, and only a median density per lot could be estimated. This design reflects practical bulk rearing conditions but introduces variability that must be acknowledged when interpreting treatment effects. The lack of container standardization directly influenced larval density, a critical parameter in BSFL production systems. Larval density affects substrate degradation dynamics, physicochemical conditions, and nutrient allocation within the larvae. High densities can accelerate substrate consumption, intensify competition for resources, and induce stress or transient starvation, potentially reducing crude fat content and altering other macronutrient fractions [21]. Variations in density among batches may therefore have contributed to asynchronous developmental patterns and to differences observed in proximate composition, phenolic accumulation, antioxidant activity, and mycotoxin levels. The experimental design was developed to approximate pilot-scale rearing conditions rather than strictly standardized laboratory units. Although container sizes varied and precise larval density control was not feasible, the proportional inclusion of agro-industrial by-products was maintained across treatments to ensure dietary comparability. This approach enhanced external validity by assessing substrate performance under practical rearing conditions, despite increased biological variability. Another limitation was the absence of the systematic quantification of survival rate and individual growth performance indicators, such as the final body weight. Due to the large-scale rearing design and the substantial larval populations per treatment, analyses focused primarily on group-level biomass yield and compositional outcomes rather than individual-based performance metrics. While this approach is appropriate for assessing substrate valorization under production conditions, it restricts the evaluation of the potential growth inhibition or toxicological effects associated with substrate composition, including phenolic exposure.

The results demonstrated clear asynchronous development among BSFL populations, with most groups reaching the prepupal stage under comparable environmental conditions. However, larvae reared on 20% APB and PPB showed two distinct developmental periods, indicating substrate-related variability [72]. Inherent biological variation and micro-environmental heterogeneity within containers may have further contributed to this divergence [73]. Differences in the developmental stage at harvest likely affected metabolic activity, nutrient allocation, antioxidant capacity, and mycotoxin accumulation, thereby limiting strict comparability among treatments.

Finally, limited biological replication further reduced the statistical robustness of the dataset. Differences in developmental timing among batches necessitated sampling at different time points, decreasing statistical power and increasing variability. As a result, the statistical analyses should be interpreted as exploratory rather than confirmatory. These constraints limit the generalizability of the results and highlight the need for future investigations incorporating increased biological replication, standardized container dimensions, controlled larval density, synchronized harvesting, and systematic growth and survival assessments.

Despite these limitations, the primary objective of this study, namely to characterize BSFL reared on phenolic-rich agro-industrial substrates and to assess the feasibility of using agricultural by-products to obtain phenolic-enriched insect biomass, was achieved.

## 5. Conclusions

This study underlines that phenolic-rich agro-industrial by-products exert strong and substrate-specific effects on the development, composition, antioxidant potential, and safety profile of BSFL. While most populations followed a typical developmental trajectory, larvae reared on 20% apple and potato peel showed asynchronous progression, underscoring the sensitivity of larval growth to substrate composition. The proximate profiles revealed pronounced nutritional modulation, with apple and beetroot peels enhancing protein deposition at higher inclusion levels, whereas excessive potato peel reduced both protein and lipid accumulation. Mineral and carbohydrate dynamics further confirmed that nutrient assimilation is highly dependent on the chemical composition and inclusion rate of each by-product.

The phenolic and flavonoid deposition in the larvae followed distinct and non-linear patterns: low-level potato peel maximized phenolic content, whereas high-level apple peel yielded the greatest flavonoid accumulation. These trends were most clearly reflected in antioxidant measurements, where treatments with elevated TPC/TFC consistently displayed superior antioxidant activity via the CUPRAC, DPPH, and ABTS methods. Colorimetric changes mirrored these biochemical shifts, indicating that dietary phenolics actively modulate larval pigmentation pathways. In contrast, rheological properties and mycotoxin accumulation were driven more by substrate-specific characteristics rather than by phenolic magnitude; viscosity was strongly influenced by beetroot-derived pigments, while ZEA levels varied widely, highlighting the divergent metabolic handling of individual toxins in BSFL. The findings of this study should be interpreted in light of the methodological constraints, particularly the limited biological replication, variability in container standardization, and asynchronous larval development. These factors likely contributed to inter-batch variability and reduced statistical power, thereby limiting strict comparability among treatments. Future research employing fully standardized rearing conditions, synchronized harvesting, and expanded replication will be essential to validate and refine the present observations.

Collectively, these findings show that phenolic-rich by-products do not exert uniform effects on BSFL but instead shape larval physiology through complex interactions between the substrate’s composition, phenolic profile, and inclusion level. Understanding these substrate-dependent responses is essential for designing optimized rearing formulations, upscaling methods and technological transfer, which balance nutritional enhancement, functional properties, and food/feed safety in BSFL production systems.

## Figures and Tables

**Table 1:** Experimental design for rearing BSFL, showing the substrate type and the percentage of phenolic-rich agro-industrial by-products added to the basic chicken feed (BBP—beetroot peel by-products, ABP—apple peel by-products, and PBP—potato peel by-products).

Experimental Groups	2%	10%	20%
Apple peel—by-products	ABP_2%_	ABP_10%_	ABP_20%_
Beetroot peel—by-products	BBP_2%_	BBP_10%_	BBP_20%_
Potato peel—by-products	PBP_2%_	PBP_10%_	PBP_20%_

**Table 2:** The number of days after the experiment began that BSFL needed to reach the prepupal stage was measured for each group. The larvae were raised on different phenolic-rich agro-industrial substrates with inclusion levels of 2%, 10%, and 20% (BBP—beetroot peel byproducts, ABP—apple peel byproducts, and PBP—potato peel byproducts).

Experimental Groups	ABP_2%_	ABP_10%_	ABP_20%_	BBP_2%_	BBP_10%_	BBP_20%_	PBP_2%_	PBP_10%_	PBP_20%_	Control
Slaughtering Time (Days After Start)	32	32	(24–32)	20	(22–32)	23	32	32	(20–32)	23

**Table 3:** Proximate composition (%) of BSFL reared on phenolic-rich agro-industrial substrates at different inclusion levels (2%, 10%, and 20%).

Exp. Groups	Ash	Protein	Moisture	Fat	Nitrogen Free Extract
ABP_2%_	17.88 ± 2.16 ^ns^	36.57 ± 7.62 ^ns^	8.45 ± 0.03 ^ns^	14.47 ± 1.90 ^ns^	22.60 ± 7.33 *
ABP_10%_	18.14 ± 3.47 ^ns^	36.8 ± 2.95 ^ns^	13.45 ± 3.22 ^ns^	18.04 ± 9.17 ^ns^	13.56 ± 11.87 ^ns^
ABP_20%_	12.51 ± 2.29 ^ns^	40.05 ± 1.37 ^ns^	16.21 ± 0.33 ^ns^	15.87 ± 6.88 ^ns^	15.34 ± 5.61 ^ns^
BBP_2%_	18.30 ± 8.87 ^ns^	34.84 ± 5.40 ^ns^	18.92 ± 7.48 *	9.57 ± 2.53 *	18.35 ± 6.54 ^ns^
BBP_10%_	13.79 ± 1.46 ^ns^	35.48 ± 4.26 ^ns^	9.02 ± 6.01 ^ns^	11.34 ± 6.13 ^ns^	30.34 ± 14.94 ***
BBP_20%_	13.66 ± 0.65 ^ns^	40.09 ± 3.94 ^ns^	6.66 ± 2.02 ^ns^	14.91 ± 0.17 ^ns^	24.64 ± 1.08 *
PBP_2%_	24.94 ± 4.78 ***	33.86 ± 2.94 ^ns^	9.06 ± 0.44 ^ns^	18.2 ± 4.44 ^ns^	13.91 ± 2.156 ^ns^
PBP_10%_	17.57 ± 6.31 ^ns^	37.63 ± 4.90 ^ns^	12.98 ± 3.50 ^ns^	11.39 ± 0.24 ^ns^	20.41 ± 2.333 ^ns^
PBP_20%_	21.22 ± 7.29 *	30.89 ± 6.03 ^ns^	14.04 ± 9.34 ^ns^	6.70 ± 2.36 ***	27.13 ± 10.451 ***
Control	12.71 ± 1.25	37.14 ± 2.11	13.88 ± 0.28	17.75 ± 1.32	18.51 ± 1.48

Values are expressed as mean ± standard deviation (SD) of two independent replicates (*n* = 2). Statistical analysis was performed using two-way ordinary ANOVA followed by Dunnett’s multiple comparison test to evaluate differences between each treatment and the control group. Statistical significance is indicated as ns (*p* > 0.05), * *p* < 0.05, *** *p* < 0.001. BBP—beetroot peel byproducts, ABP—apple peel byproducts, and PBP—potato peel byproducts.

**Table 4:** Colorimetric parameters (CIELAB L*, a*, and b*) and total color difference (ΔE) of BSFL reared on agro-industrial substrates at different inclusion levels (2%, 10%, and 20%).

Exp. Groups	±L*	±a*	±b*	ΔE
ABP_2%_	30.83 ± 2.37 **	4.39 ± 0.80 ^ns^	16.60 ± 5.39 ^ns^	13.73 ± 2.38 **
ABP_10%_	36.62 ± 3.37 ***	5.51 ± 1.67 ^ns^	17.42 ± 2.20 ^ns^	18.14 ± 3.25 ***
ABP_20%_	23.09 ± 5.32 ^ns^	6.97 ± 1.35 ^ns^	15.15 ± 4.13 ^ns^	6.34 ± 3.46 ^ns^
BBP_2%_	24.51 ± 9.80 ^ns^	5.30 ± 0.37 ^ns^	13.03 ± 3.04 ^ns^	9.17 ± 5.32 ^ns^
BBP_10%_	39.76 ± 5.71 ***	5.53 ± 0.36 ^ns^	17.04 ± 0.68 ^ns^	21.44 ± 5.23 ***
BBP_20%_	32.24 ± 0.53 **	4.86 ± 0.42 ^ns^	16.40 ± 0.35 ^ns^	14.82 ± 0.45 ***
PBP_2%_	43.30 ± 1.39 ***	5.83 ± 0.73 ^ns^	17.31 ± 2.59 ^ns^	24.60 ± 1.23 ***
PBP_10%_	35.53 ± 5.85 ***	6.18 ± 0.02 ^ns^	16.74 ± 0.26 ^ns^	16.87 ± 5.55 ***
PBP_20%_	29.02 ± 3.94 *	5.73 ± 0.64 ^ns^	13.53 ± 0.29 ^ns^	10.71 ± 3.88 *
Control	19.16 ± 1.25	8.96 ± 0.38 ^ns^	14.74 ± 0.69 ^ns^	-

±L*, ±a*, and ±b* represent the standard deviations (SD; *n* = 2) of the CIELAB color coordinates, where L* indicates lightness, a* the red–green axis, and b* the yellow–blue axis. ΔE denotes the overall color difference calculated from these parameters. Statistical analysis was performed using two-way ordinary ANOVA followed by Dunnett’s multiple comparison test to assess differences between each treatment and the control group. Statistical significance is indicated as ns (*p* > 0.05), * *p* < 0.05, ** *p* < 0.01, and *** *p* < 0.001.

**Table 5:** Mycotoxin analysis results. AFB1 and ZEA concentrations in BSFL reared on agro-industrial substrates at 2%, 10%, and 20% inclusion levels.

Exp. Groups	AFB1 (µg/kg)	ZEA (µg/kg)
ABP_2%_	2.37 ± 0.22	178.39 ± 11.37
ABP_10%_	2.55 ± 0.47	170.14 ± 135.02
ABP_20%_	2.47 ± 0.07	203.85 ± 61.03
BBP_2%_	2.53 ± 0.31	208.13 ± 75.68
BBP_10%_	2.34 ± 0.40	254.72 ± 10.91
BBP_20%_	2.26 ± 0.21	202.14 ± 105.84
PBP_2%_	2.52 ± 0.53	256.49 ± 147.14
PBP_10%_	2.32 ± 0.07	105.03 ± 38.35
PBP_20%_	2.59 ± 0.33	248.26 ± 138.53
Control	2.87 ± 0.25	109.04 ± 0.29

## Data Availability

The data presented in this study are available within the article. Additional data supporting the findings of this study are available from the corresponding author upon reasonable request.

## Abbreviations

The following abbreviations are used in this manuscript:

ABP	Apple-containing by-products
BBP	Beetroot-containing by-products
PBP	Potato-containing by-products
BSFL	Black Soldier Fly larvae
